# Exploring the feasibility and implications of cranioencephalic computed tomography in HER2-positive breast cancer: A pilot study

**DOI:** 10.1016/j.heliyon.2024.e33886

**Published:** 2024-06-28

**Authors:** Ana Fortuna, Paulo Luz, Magda Cordeiro, Beatriz Gosalbez, Elsa Reis Campoa, Melanie Claudino, Pedro Alves, João G. Costa, Ana S. Fernandes, Cidália Pinto

**Affiliations:** a Oncology Department, Unidade Local de Saúde do Algarve, EPE, 8000, Faro, Portugal; b Faculty of Medicine, University of Porto, 4200, Porto, Portugal; c CBIOS - Universidade Lusófona's Research Center for Biosciences & Health Technologies, 1749, Lisboa, Portugal; d Department of Biomedical Sciences, University of Alcalá, 28805, Madrid, Spain; e Oncology Center, Hospital Particular do Algarve, Grupo HPA Saúde, 8005, Faro, Portugal; f Oncology Department, Unidade Local de Saúde do Algarve, EPE, 8500, Portimão, Portugal; g Radiology Department, Unidade Local de Saúde do Algarve, EPE, 8000, Faro, Portugal

**Keywords:** HER2-Positive breast cancer, Brain metastases, Cranial computed tomography, Staging, Screening

## Abstract

**Background:**

Breast cancer has several subtypes, including HER2-positive breast cancer, which is characterized by overexpression of human epidermal growth factor receptor 2 (HER2), aggressiveness, poor prognosis, and high risk of recurrence and metastasis. Brain metastases are a common complication of HER2-positive breast cancer, but brain imaging is not included in the initial staging of this disease. This prospective pilot study aimed to evaluate the usefulness of brain computed tomography (CT) in the initial staging of HER2-positive breast cancer.

**Patients and methods:**

Fifty-eight patients were enrolled and demographic, clinical, and breast cancer-specific data were collected after the informed consent and ethical approval were obtained.

**Results:**

A descriptive analysis was performed. The median age of the patients was 55 years, and the majority had good performance status. Brain CT scans were performed at diagnosis, and no brain metastases were detected in early-stage patients. However, brain CT identified brain metastases in one advanced-stage patient with clinical suspicion.

**Conclusions:**

This study suggests that brain CT may have limited utility in the initial staging of early HER2-positive breast cancer, while it could be a valuable tool in advanced cases. Further research is needed, including a higher number of patients to identify those with high risk, which may benefit from brain imaging.

## Highlights

• Prospective data regarding the use of brain imaging for early staging in patients with HER2+ breast cancer are limited.
• Brain CT detected no metastases in asymptomatic patients, mostly those with early-stage tumors.
• Research should focus on identifying high-risk patients and exploring the benefits of early detection of brain metastases.

## 1 Introduction

Breast cancer is a complex disease with a variety of subtypes, including HER2-positive breast cancer. This subtype is characterized by the overexpression of the human epidermal growth factor receptor 2 (HER2) protein, which is historically associated with tumor aggressiveness and poor prognosis, with an increased risk of recurrence, metastasis, and mortality [1]. This oncogene is amplified in approximately 20 % of breast cancers [2].

HER2-positive and triple-negative breast cancers metastasize to the brain more frequently than luminal cancers [3,4] and this occurrence has been increased because patients are living longer [5]. According to the International Breast Cancer Study Group trials, in early-stage HER2-positive breast cancer, the 10-year cumulative incidence rate of central nervous system metastasis was approximately 7 % for HER2-positive tumors, compared to a rate of 3.5 % for HER2-negative tumors [6]. Other studies have reported 12-month, 24-month, and 12-year cumulative incidences of brain metastases of 0.6 %, 2 %, and 10 %, respectively [4,7]. During follow-up, brain relapse occurs in approximately 2 % of patients initially diagnosed with early-stage disease [8,9]. Otherwise, about 20–50 % of patients with advanced HER2-positive breast cancer patients are diagnosed with brain metastases, significantly reducing their quality of life and overall survival [3,10].

Although brain metastases are a common complication of HER2-positive breast cancer, current guidelines do not recommend brain imaging screening in clinical practice. Recent guidelines suggest that it may have a potential role only in advanced stages [11]. However, to date, no study has evaluated the role of brain computed tomography (CT) in the initial staging of this subtype of breast cancer. Ongoing clinical trials (NCT03881605, NCT03617341, NCT04030507) are investigating the potential utility of proactive screening strategies in selected patients at increased risk for brain metastases (BM), such as those with metastatic HER2-positive breast cancer.

This raises the question of whether the use of brain imaging at initial diagnosis can influence patient management by detecting asymptomatic brain metastases. Thus, this prospective study aimed to evaluate the usefulness of brain CT in the staging of patients with HER2-positive breast cancer.

## 2 Materials and methods

This study had the approval of the board of directors and the ethics committee of the Centro Hospitalar Universitário do Algarve (ethics approval reference number: UAIF 126/2020) on February 8, 2021. All participants enrolled in the study provided informed consent after receiving a comprehensive briefing of the study objectives, procedures, potential risks, and benefits. Participants were provided with ample opportunity to ask questions and clarify any concerns before voluntarily providing written informed consent.

All data collection and handling procedures strictly adhered to applicable privacy regulations and institutional policies and ensured the confidentiality and protection of patient data. The anonymity of the participants and confidentiality of the data were strictly maintained.

Fifty-eight patients consented to the inclusion of brain CT in initial breast cancer staging. Demographic and clinical information including sex, age at diagnosis and menopausal status were collected. Tumor marker (CA15.3), hormone receptors, antigen kiel 67 (ki67) status, and breast cancer information including histology grade, presence of lymphovascular or perineural invasion, presence of lymphocytic infiltrate or microcalcifications were obtained from the clinical and staging records of each patient.

Descriptive analysis was used to characterize the main characteristics of the population studied. Statistical analysis was performed using SPSS software (v29). Medians are reported to provide a comprehensive overview of the demographic and clinical profiles of patients in our study population, along with corresponding measures of variability.

## 3 Results

During the study period, a total of 58 patients diagnosed with HER2-positive breast cancer underwent brain CT scan for screening for brain metastases at the time of diagnosis. The patients' median age was 55 years (range, 33–87 years), and the majority had a good performance status (ECOG grade = 0) (n = 45, 77.6 %). Baseline patient characteristics showed a gender distribution of 98.3 % female and 1.7 % male (Table 1).

**Table 1:** Characteristics of study participants.

	Total Patients (n = 58)
**Gender, % (n)**	
Male	1.7 (1)
Female	98.3 (57)
**Median age, years**	55 (13.5)
ECOG PS*, % (n)	
0	77.6 (45)
1	17.2 (10)
Missing	8.6 (5)
**Menopausal Status, % (n)**	
Pre-menopausal	35.1 (20)
Post-menopausal	61.4 (35)
Missing	3.5 (2)
**CA 15.3, UI/L (n)**	18 (12)

Data are expressed as median (SD) and % (n) for continuous and categorical variables, respectively. Abbreviations: CA, cancer antigen; ECOG PS, Eastern Cooperative Oncology Group Performance Status scale.

Regarding the primary tumor characteristics, 19 patients (32.8 %) had HR-negative tumors, and 25 patients (43.1 %) had grade 3 tumors. Tumor-infiltrating lymphocytes (TILs) were present in 24 patients (41.4 %). Detailed information on the oncological characteristics of the study participants can be found in Table 2.

**Table 2:** Oncological characteristics of study participants.

Patient characteristics (N = 58)	N (%)
**HER2 status**	
3+	53 (91.4)
2+, confirmed by SISH	5 (8.6)
**Estrogen receptors**	
Negative	21 (36.2)
Positive	37 (63.8)
**Progesterone receptors**	
Negative	32 (55.2)
Positive	26 (44.8)
**Ki67 %**	
<20	15 (25.9)
20–40	23 (39.7)
>40	20 (34.5)
**Histologic grade**	
Well-differentiated (G1)	6 (10.3)
Moderately differentiated (G2)	26 (44.8)
Undifferentiated (G3)	25 (43.1)
Missing	1 (1.7)
**Lymphovascular invasion**	
Absent	35 (60.3)
Present	10 (17.2)
Missing	3 (5.1)
**Perineural permeation**	
Absent	28 (48.3)
Present	14 (24.1)
Unknown	16 (27.6)
**Tumor-infiltrating lymphocyte**	
Absent	18 (31.0)
Present	24 (41.4)
Unknown	16 (27.6)
**Staging at diagnosis**	
**Tumor Size**	
T1	13 (22.4)
T2	33 (56.9)
T3	8 (13.8)
T4	4 (6.9)
**Lymph node status**	
N0	27 (46.6 %)
N+	29 (50.0 %)
Unknown	2 (3.4 %)
**Distant metastasis at diagnosis**	
M0	53 (91.4 %)
M1	5 (8.6 %)

Abbreviations: HER2, human epidermal growth factor receptor 2; Ki67, Antigen Kiel 67; SISH, silver in situ hybridization.

The majority of patients had early-stage breast cancer (stage I/II/III) (n = 53, 93.4 %), while a subset of patients had metastatic disease at diagnosis (n = 5, 8.6 %).

Brain metastases were not detected in any of the patients diagnosed with early-stage breast cancer. However, a patient with metastatic disease, involving the liver and lungs, presented brain metastases at diagnosis. This patient showed neurological symptoms.

The majority of patients received neoadjuvant chemotherapy (n = 49; 84.5 %). During a median follow-up of 19.5 months, 3 patients experienced disease recurrence and progression. Brain recurrence was detected in a patient diagnosed with stage IA breast cancer, 22.5 months after the initial diagnosis. The tumor was moderately differentiated (G2), HR-positive, and had a small lymphocytic infiltrate. In addition, two patients who were initially diagnosed with metastatic disease showed brain progression, associated with symptomatic disease.

## 4 Discussion

Given the high incidence of brain metastases in HER2-positive breast cancer, it is important to assess whether adding brain imaging to the initial staging can detect asymptomatic brain metastases and thus change the treatment approach for patients. Our pilot study aimed to address this research gap in HER2-positive breast cancer staging. From what we know, this is the first study addressing this issue and including patients with early breast cancer, as most studies focus solely on the metastatic setting.

The median age of the patients in our study was 55 years, with the majority having HR + tumors, as well as early or locally advanced HER2+ tumors. Additionally, 57.1 % of the tumors exhibited some level of tumor-infiltrating lymphocytes. These demographic and clinical characteristics closely align with the global data and averages reported in the literature [12,13]. In our study, no BM were detected on brain CT scans in asymptomatic patients. Most of the patients included in this study had early-stage disease, an area with limited published data on the role of BM screening. According to our pilot study, brain CT was unable to detect BM at the time of diagnosis of early-stage HER2-positive breast cancer.

A large retrospective study that analyzed 231,684 patients diagnosed with BM and newly diagnosed breast cancer showed 9.4 % of cases were HR-positive and HER2-positive, and 4.1 % were HR-negative and HER2-positive. The median survival for patients with the HR-positive/HER2-positive subtype was 21.0 months, while for those with the HR-negative/HER2-positive subtype, it was 10.0 months [12]. In this study, the incidence of BM was 0.61 % among HR+/HER2+ breast cancer patients and 1.09 % among HR-/HER2+ patients at the time of diagnosis. It's worth noting that these patients exhibited neurological symptoms at diagnosis, which might underestimate the true incidence of BM [12].

There are no data available about screening for BM in early breast cancer because in most studies the incidence of BM at diagnosis is typically detected based on the presence of symptoms rather than proactive brain imaging.

One of the major limitations of our study was the small sample size. Furthermore, more effective imaging techniques are available for characterizing brain lesions than brain CT. Brain magnetic resonance imaging (MRI) is the most suitable choice for this evaluation, despite its higher cost and limited accessibility. However, considering that very few patients experienced brain relapse during the follow-up, MRI would probably not alter the results. Despite our small sample size, the studied population is reflective of the real-world scenario, particularly in countries with screening programs and a public health system. This provides a level of validation to our study population, allowing for a pilot extrapolation of results to a broader context.

Our prospective pilot study indicates that brain CT may not have a substantial role in the initial staging of early HER2-positive breast cancer. Considering the low incidence of BM at the time of diagnosis in this context, coupled with the anxiety induced by the examination and the potential financial burden, as well as the radiation exposure to the brain, it raises questions about its utility in this setting. Due to the limited number of patients with metastatic disease included, it is not possible to draw conclusive statements regarding this setting. However, brain screening may be beneficial in this context, as early diagnosis may allow for more conservative local therapies, such as radiosurgery, to be used [14,15]. Retrospective data have shown improved outcomes and quality of life for patients with detected asymptomatic lesions [16,17]. This positive impact may be attributed to the option of choosing radiosurgery over open surgery as a treatment approach. In addition, new treatment options with demonstrated brain activity, such as tucatinib, trastuzumab deruxtecan, and pyrotinib, are now available and may be used even in the presence of active lesions [[18], [19], [20]].

Future research should focus on identifying early-stage HER2+ breast cancer patients at high risk of developing BM and the regular use of BM screening during the follow-up. Numerous studies have consistently identified higher presenting stage, histological grade, tumor size, Ki67 expression, and nodal involvement as independent risk factors for BM [21]. In this scenario, incorporating genetic data may also provide deeper insights into the risk of metastatic progression. In the ever-evolving landscape of medical advancements, the integration of AI and machine learning algorithms stands as a promising avenue for the early identification of HER2+ breast cancer patients at an elevated risk of brain relapse [22].

Considering the high incidence of BM, attention should also be paid to the metastatic setting and the use of brain imaging screening during treatment. Also, it is essential to evaluate not only the incidence but also the impact of early diagnosis on both quality of life and overall survival. Additionally, considerations must extend to the mid/long-term effects of radiotherapy and the potential side effects associated with transitioning to treatment options demonstrating cerebral activity.

## 5 Conclusions

In conclusion, our pilot study suggests that brain CT may not significantly contribute to the initial staging of early HER2-positive breast cancer. Future studies should focus on the metastatic setting and assess the impact of early diagnosis on quality of life and survival. A comprehensive understanding of these aspects will guide more effective strategies for early detection and improved patient outcomes and quality of life.

## Ethical statement

The study was conducted in accordance with the Declaration of Helsinki, and approved by the Institutional Review Board and the Ethics Committee of Centro Hospitalar Universitário do Algarve (protocol code UAIF 126/2020, February 08, 2021). Written informed consent was obtained from all subjects for study participation and data publication.

## Data availability statement

The data are not publicly available due to ethical constraints.

## Funding

This research did not receive any specific funding.

## CRediT authorship contribution statement

**Ana Fortuna:** Writing – review & editing, Writing – original draft, Investigation, Conceptualization. **Paulo Luz:** Writing – review & editing, Writing – original draft. **Magda Cordeiro:** Investigation, Conceptualization. **Beatriz Gosalbez:** Writing – review & editing, Writing – original draft, Investigation. **Elsa Reis Campoa:** Writing – review & editing, Investigation. **Melanie Claudino:** Writing – review & editing, Investigation. **Pedro Alves:** Writing – review & editing, Investigation. **João G. Costa:** Writing – review & editing, Investigation. **Ana S. Fernandes:** Writing – review & editing. **Cidália Pinto:** Writing – review & editing.

## Declaration of competing interest

The authors declare that they have no known competing financial interests or personal relationships that could have appeared to influence the work reported in this paper.
